# Prevalence of Hypercalcaemia in a Renal Transplant Population: A Single Centre Study

**DOI:** 10.1155/2016/7126290

**Published:** 2016-07-14

**Authors:** Tony Amin, P. Toby Coates, Jeffrey Barbara, Paul Hakendorf, Nazmul Karim

**Affiliations:** Central Northern Adelaide Renal and Transplantation Services, Royal Adelaide Hospital, Adelaide, SA 5000, Australia

## Abstract

*Introduction*. Postrenal transplant bone disease is a significant problem. Factors influencing postrenal transplant bone status include high dose acute and low dose long-term steroid use, persistent hypercalcaemia, and graft failure. In this study, we aimed to determine the prevalence of hypercalcaemia and to evaluate the risk factors for postrenal transplant hypercalcaemia in long-term renal transplant patients at our centre.* Methods*. This is a biochemical audit in which we studied renal transplant recipients from the Central Northern Adelaide Renal Transplant Services, South Australia. Inclusion criteria include kidney transplant patients with functioning graft since 1971 and at least 3 months after transplantation at the time of analysis. Hypercalcaemia was defined as persistently elevated serum corrected calcium greater than or equal to 2.56 mmol/L for three consecutive months.* Results*. 679 renal transplant recipients with a functioning graft were studied and 101 were hypercalcaemic between March 2011 and June 2011 (15%). 60% of the hypercalcaemic patients were male and 40% were female, with chronic glomerulonephritis (39%) being the commonest cause of their end stage kidney disease (ESKD). Prevalence was similar in those that had haemodialysis and peritoneal dialysis pretransplantation. Hypercalcaemia in the renal transplant population was not secondary to suboptimal allograft function but secondary to pretransplantation hyperparathyroidism with persistent high parathyroid hormone (PTH) levels after transplantation.* Conclusion*. There is a high prevalence of hypercalcaemia (15%) in renal transplant recipients. The predominant cause for hypercalcaemia is pretransplantation hyperparathyroidism. The magnitude of pretransplantation hyperparathyroidism is the major determinant for long-term parathyroid function rather than graft function or pretransplantation duration on dialysis or mode of dialysis.

## 1. Introduction

Renal transplant bone disease is a significant problem. Factors influencing postrenal transplant bone disease include high dose acute and low dose long-term steroid use, persistent hypercalcaemia, and graft function. The prevalence of hypercalcaemia after renal transplant may vary from 5 to 50% [1]. The clinical impact of hypercalcaemia was debatable, but recent evidence shows that postrenal transplant hypercalcaemia has an impact on graft outcome and hypercalcaemia is a risk factor for vascular calcification [2, 3]. Posttransplant hypercalcaemia could also be an independent risk factor for acute pancreatitis in the renal transplant population [4].

The prevalence of persistent hyperparathyroidism after renal transplantation has been generally appreciated using hypercalcaemia as an index [5]. Posttransplant hypercalcaemia could be secondary to pretransplantation secondary or tertiary hyperparathyroidism and could also be secondary to recovered circulating levels of 1,25 OH_2_ vitamin D due to increased renal tubular synthesis [6, 7]. Secondary hyperparathyroidism usually improves between the first month and six months after renal transplantation with a reduction in parathyroid mass [8]. The renal function and magnitude of hyperparathyroidism whilst on dialysis could be the major determinants of long-term parathyroid function, even after postrenal transplantation [9]. Although the resolution of parathyroid function towards normal is common in postrenal transplantation, in many cases, parathyroid level could be persistently elevated after renal transplantation. This postrenal transplant hyperparathyroidism could be treated with a calcimimetic or parathyroidectomy, although posttransplant parathyroidectomy may decrease renal transplant function as PTH has a known positive regulatory effect on renal perfusion and glomerular filtration rate [10, 11]. We have recently shown that posttransplantation oral calcium therapy is an effective treatment for suppression of PTH [12]. In this study, we aimed to determine the prevalence of hypercalcaemia and to evaluate the risk factors for postrenal transplant hypercalcaemia in long-term renal transplant patients at our centre.

## 2. Methods

A biochemical audit was carried out on routine biochemical test done in outpatient clinic for an unselected renal transplant population between 1971 and 2011 from the Central Northern Adelaide Renal Transplant Service, South Australia. Inclusion criterias are live or deceased donor renal or combined renal pancreatic transplants with a functioning renal allograft in our centre and at least 3 months after transplantation at the time of analysis. Exclusion criteria included failed grafts and patients followed up in other centres. Hypercalcaemia was defined as persistently elevated serum corrected calcium greater than or equal to 2.56 mmol/L (laboratory highest range for serum calcium) for more than consecutive three months.

The following clinical data of hypercalcaemic population were registered and analyzed: age, sex, primary renal disease, dialysis duration and mode of dialysis, renal function, phosphate, and pre- and posttransplant PTH levels. Pre- or posttransplant treatment for secondary hyperparathyroidism has not been assessed here.

### 2.1. Lab Method

All the biochemical analyses for serum creatinine, calcium, and PTH had been done on Siemen's Advia chemistry system. Serum creatinine had been measured by Jaffe method using picric acid and calculation of eGFR had been done using 175 MDRD formulas. Serum calcium method is a dye binding method and intact PTH level was measured using immunoassay. Corrected calcium has been measured using albumin correction alone.

### 2.2. Statistical Analyses

All statistical analyses were performed using STATA statistical software version 12.0 (StataCorp, LP, College Station, TX, USA). Student's *t*-test was used to compare continuous variables. Chi-squared test was used for categorical variables. Cox proportional hazard regression was used for analyzing time to outcome data. As this study was initiated to review the prevalence of hypercalcaemia in long-term postrenal transplant patients, the role of control with normal postrenal transplantation calcium level was limited and has been excluded from this study and no control has been examined for this study.

## 3. Results

679 renal transplant patients with a functioning allograft were studied with spanning duration of 40 years. A total of 101 patients were found to be hypercalcaemic between March 2011 and June 2011, which represents 15% of renal transplant recipients.

### 3.1. Population

Amongst hypercalcaemic population, 61% (*n* = 62) were male and 39% (*n* = 39) were female. Mean age of hypercalcaemic population was 55 with a standard deviation (SD) of 13 (Figure 2).

Postrenal transplant duration amongst these hypercalcaemic populations varies between one and twenty-seven years. Male and female ratio in this curve is similar.

### 3.2. Primary Renal Disease

Chronic glomerulonephritis (34%) (*n* = 34) is the commonest cause of end stage renal disease (ESRD) in this hypercalcaemic population. Other causes of ESRD were reflux nephropathy 16% (*n* = 16), diabetic nephropathy 12% (*n* = 12), and polycystic kidney disease 12% (*n* = 12).

### 3.3. Duration and Mode of Dialysis

**Figure 1:**
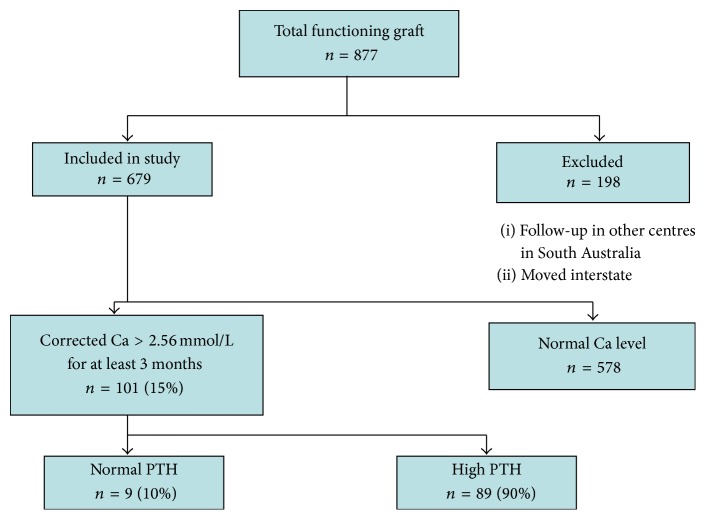
Flowchart showing prevalence of postrenal transplant hypercalcaemia and hypercalcaemic patients pretransplantation PTH level.

**Figure 2:**
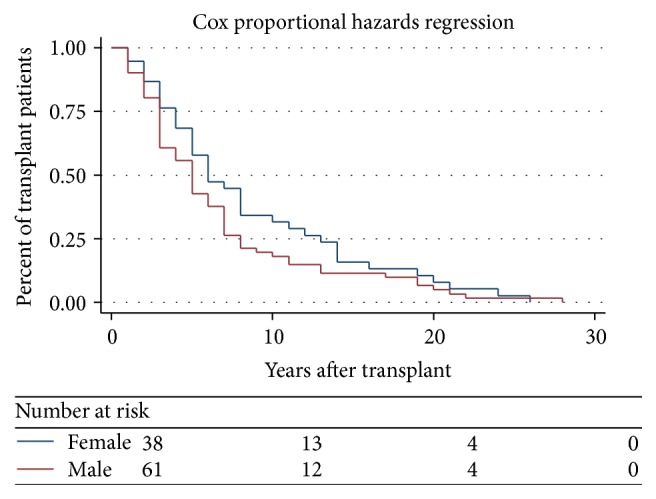
Years after transplant for male and female hypercalcaemic population.

The predominant form of renal replacement therapy amongst the hypercalcaemic population prior to transplantation was haemodialysis (HD) with 64% (*n* = 65) of the patients; 18% (*n* = 18) were on peritoneal dialysis (PD) and 8% (*n* = 7) of patients had preemptive renal transplantation. 11% (*n* = 11) of patients had experienced both modes of dialysis with transfer from PD to HD following PD failure (Figure 1).

**Figure 3:**
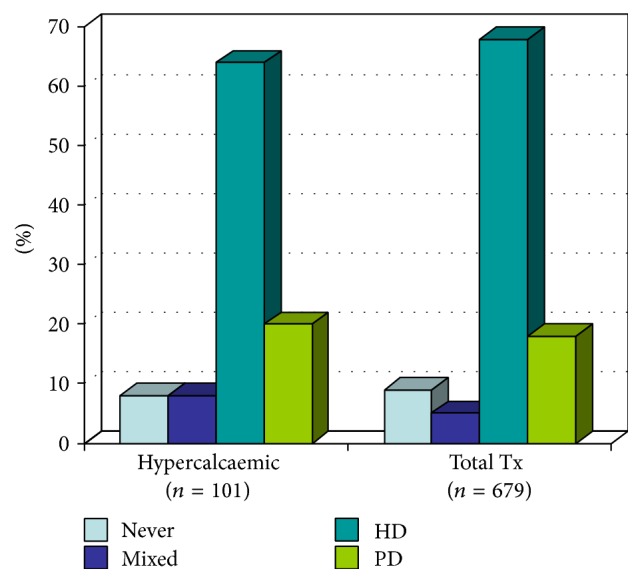
Comparison of modes of dialysis between the hypercalcaemic population and all renal transplant recipients.

Haemodialysis had been the predominant mode of dialysis amongst the hypercalcaemic group and a similar percent had also received HD in the nonhypercalcaemic group. Of all functioning grafts (*n* = 679), HD comprised 68% (*n* = 462), PD 18% (*n* = 122), and preemptive transplants 9% (*n* = 61), and mixed mode of dialysis was 5% (*n* = 34), which was similar to hypercalcaemic group (Figure 3).

**Figure 4:**
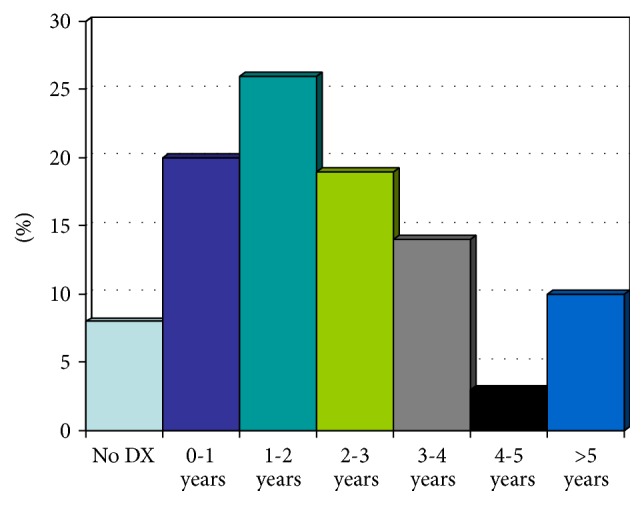
Figure showing pretransplantation duration on dialysis for hypercalcaemic patients.

Of the hypercalcaemic population, only 10% (*n* = 10) had been on dialysis for more than 5 years whereas 8% (*n* = 8) of hypercalcaemic population were never on any mode of dialysis (preemptive transplant). 20% (*n* = 21) were on dialysis for less than one year, 26% (*n* = 27) had been on dialysis only for 1-2 years, 18% (*n* = 18) were on dialysis for 2-3 years, 14% (*n* = 14) were on dialysis for 3-4 years, and 3% (*n* = 3) were on dialysis for 4-5 years (Figure 4).

The hypercalcaemic patients had median time of 2.4 years on dialysis before the renal transplantation with a standard deviation of 2.

**Figure 5:**
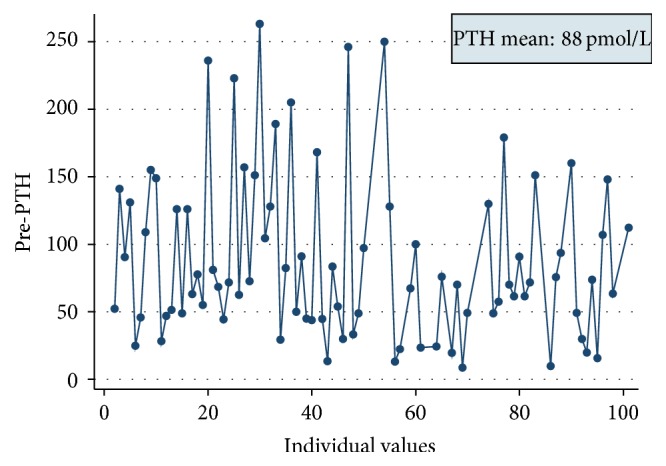
Pretransplant PTH levels for the posttransplantation hypercalcaemic population.

**Figure 6:**
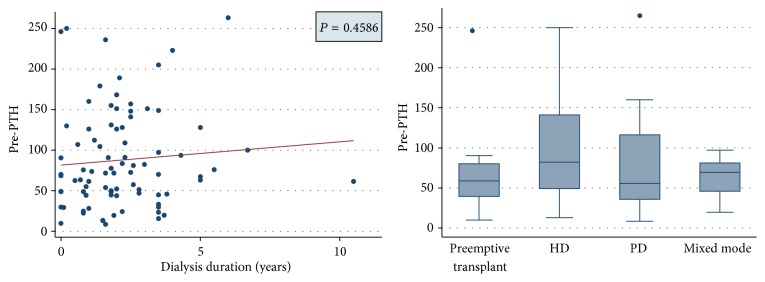
Figure showing correlation between pretransplantation PTH levels and duration and modes of dialysis.

Interestingly, the prevalence of pretransplantation hyperparathyroidism was very high (mean pretransplantation PTH: 88 pmol/L) (Figure 5) and PTH levels were similar amongst all modes of dialysis during this period and did not correlate with duration on dialysis prior to renal transplant (Figure 6).

### 3.4. Hypercalcaemia, PTH, and Graft Function

The prevalence of hypercalcaemia was highest in the group of patients with eGFR between 30 and 60 mL/min (50%) (*n* = 50) and 44% (*n* = 44) had eGFR > 60. Incidence reduced to 6% (*n* = 7) in eGFR < 30 population. None of the hypercalcaemic patients had eGFR < 15 and all patients had serum phosphates (PO4) within normal limit.

**Figure 7:**
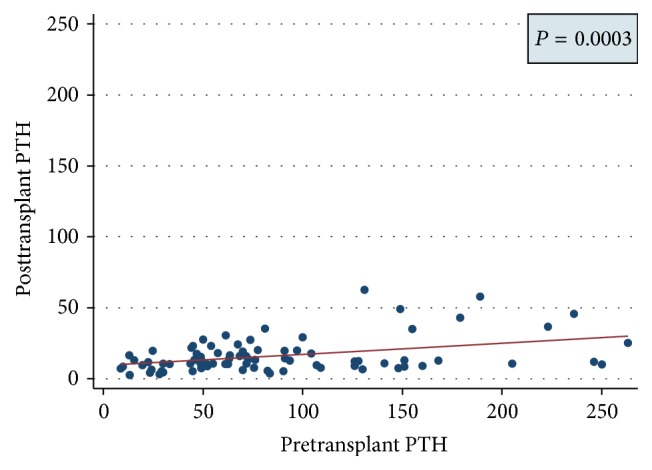
Figure showing correlation between pre- and posttransplant PTH levels (*P* value 0.0003; CI 95%).

The posttransplant recovery of parathyroid level was directly proportional to pretransplantation parathyroid levels and posttransplant PTH levels were independent of their graft function (Figure 7).

## 4. Discussion

In order to obtain a better insight into the prevalence of posttransplant hypercalcaemia in our centre, we performed a retrospective observational study of 679 patients who received a primary renal allograft between 1971 and 2011 and still have a functioning renal allograft. The prevalence of hypercalcaemia was 15% in our centre. Interestingly, it was also found that the patients with predominant pretransplant hyperparathyroidism were mostly suffering from posttransplantation hypercalcaemia. We also found a significant correlation between pre- and posttransplant PTH levels that was similar to the findings of Bonarek et al. [8].

Renal transplant is the optimal treatment for patients with end stage kidney disease (ESKD). It improves the quality of life and survival and reduces metabolic complications. In terms of bone loss, almost all patients develop low bone turnover or a mixed renal osteodystrophy six months after transplantation in contrast to patients with high turnover disease at transplantation. Patients with adynamic bone disease appear to improve after renal transplantation [13, 14]. High parathyroid levels before renal transplantation might induce a significant bone loss within the first months after transplantation and parathyroid function improvement observed in the first 6 months is mainly due to reduction in parathyroid functional mass [8, 15]. Another interesting observation in our study was that the hypercalcaemic patients have a higher posttransplantation PTH than the recommended normal range. We believe our study will lead to further study in posttransplant hypercalcaemic patients to evaluate and compare posttransplant PTH levels between the normocalcaemic and hypercalcaemic group, which will help determining postrenal transplant recommended PTH level and will guide us regarding the management of posttransplantation hyperparathyroidism leading to hypercalcaemia. A pilot study has suggested that short-term calcium replacement after transplantation may improve initial acute bone loss and also improve posttransplant PTH levels [12].

**Figure 8:**
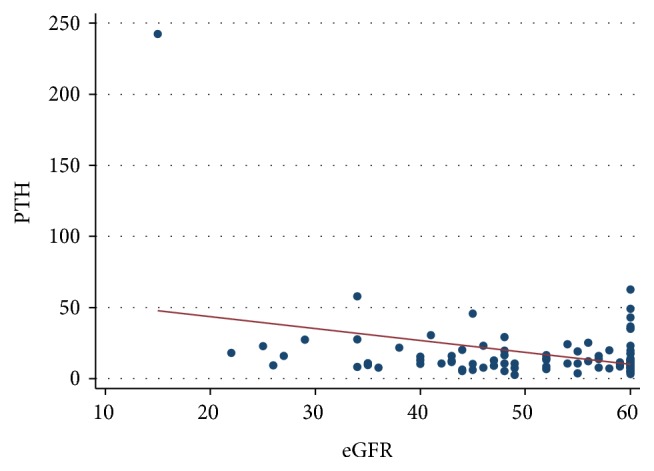
Posttransplant PTH levels are independent of posttransplant allograft function.

Our study also reveals that hypercalcaemia is more common in male subjects but does not relate to the duration of dialysis or posttransplant duration. The prevalence was also similar with different modes of dialysis. No significant difference in the prevalence of hypercalcaemia in the renal transplant population between optimal and suboptimal allograft function and also no significant differences in posttransplant parathyroid function were observed between patients with optimal and suboptimal allograft function (Figure 8). Significant pretransplant hyperparathyroidism and persistently high posttransplant PTH levels were observed in the hypercalcaemic population. The pretransplantation PTH levels were high with all modes of dialysis and did not vary with the duration of dialysis pretransplantation. As shown by Bonarek et al. [8], there was also a strong correlation between pre- and posttransplant PTH levels in this study.

There are limitations of this biochemical audit. As this audit included patients with large variation in time since transplantation, pretransplant PTH levels for 14 patients were not possible to retrieve at the time of analysis. We also did not consider management of pre- or posttransplant hyperparathyroidism during our audit that may have impact on prevalence of hypercalcaemia as well as PTH levels. In this relation, our future endeavor is to analyze long-term cardiovascular complications in the hypercalcaemic population and compare parameters between hypercalcaemic and normocalcaemic population.

In conclusion, the prevalence of hypercalcaemia in the renal transplant recipients is 15% in our centre. The predominant cause of hypercalcaemia is pretransplant hyperparathyroidism. The magnitude of pretransplantation hyperparathyroidism is the major determinant for long-term parathyroid function rather than graft function or pretransplant duration or mode of dialysis. Thus, in order to prevent posttransplant hypercalcaemia and improve metabolic outcome after renal transplantation, efforts should be made to control hyperparathyroidism effectively during the pretransplantation period.
